# Identification of novel CDK9 and Cyclin T1-associated protein complexes (CCAPs) whose siRNA depletion enhances HIV-1 Tat function

**DOI:** 10.1186/1742-4690-9-90

**Published:** 2012-10-30

**Authors:** Rajesh Ramakrishnan, Hongbing Liu, Hart Donahue, Anna Malovannaya, Jun Qin, Andrew P Rice

**Affiliations:** 1 Department of Molecular Virology & Microbiology, Baylor College of Medicine, Houston, Texas, USA; 2 Department of Molecular and Cellular Biology, Baylor College of Medicine, Houston, Texas, USA

## Abstract

**Background:**

HIV-1 Tat activates RNA Polymerase II (RNAP II) elongation of the integrated provirus by recruiting a protein kinase known as P-TEFb to TAR RNA at the 5^′^ end of nascent viral transcripts. The catalytic core of P-TEFb contains CDK9 and Cyclin T1 (CCNT1). A human endogenous complexome has recently been described – the set of multi-protein complexes in HeLa cell nuclei. We mined this complexome data set and identified 12 distinct multi-protein complexes that contain both CDK9 and CCNT1. We have termed these complexes CCAPs for CDK9/CCNT1-associated protein complexes. Nine CCAPs are novel, while three were previously identified as Core P-TEFb, the 7SK snRNP, and the Super-Elongation Complex. We have investigated the role of five newly identified CCAPs in Tat function and viral gene expression.

**Results:**

We examined five CCAPs that contain: 1) PPP1R10/TOX3/WDR82; 2) TTF2; 3) TPR; 4) WRNIP1; 5) FBXO11/CUL1/SKP1. SiRNA depletions of protein subunits of the five CCAPs enhanced Tat activation of an integrated HIV-1 LTR-Luciferase reporter in TZM-bl cells. Using plasmid transfection assays in HeLa cells, we also found that siRNA depletions of TTF2, FBXO11, PPP1R10, WDR82, and TOX3 enhanced Tat activation of an HIV-1 LTR-luciferase reporter, but the depletions did not enhance expression of an NF-κB reporter plasmid with the exception of PPP1R10. We found no evidence that depletion of CCAPs perturbed the level of CDK9/CCNT1 in the 7SK snRNP. We also found that the combination of siRNA depletions of both TTF2 and FBXO11 sensitized a latent provirus in Jurkat cells to reactivation by sub-optimal amounts of αCD3/CD28 antibodies.

**Conclusions:**

Our results identified five novel CDK9/CCNT1 complexes that are capable of negative regulation of HIV-1 Tat function and viral gene expression. Because siRNA depletions of CCAPs enhance Tat function, it is possible that these complexes reduce the level of CDK9 and CCNT1 available for Tat, similar to the negative regulation of Tat by the 7SK snRNP. Our results highlight the complexity in the biological functions of CDK9 and CCNT1.

## Background

A critical step in the HIV-1 replication cycle is transcription of the integrated provirus by RNA polymerase II (RNAP II). Although RNAP II initiates transcription from the HIV-1 LTR at a relatively high basal rate, the polymerase stalls after synthesis of a short transcript due to the action of two negative elongation factors, NELF and DSIF, which associate with the RNAP II complex and inhibit elongation [reviewed in [1-4]]. Pausing of RNAP II elongation is also seen in cellular genes and serves as a quality control mechanism to enable efficient capping of the 5^′^ end of the mRNA [5,6]. In the case of HIV-1, the viral Tat protein recruits a cellular protein kinase complex termed P-TEFb to the TAR RNA element that forms at the 5^′^ ends of the nascent viral transcript. The catalytic component of P-TEFb is composed of CDK9 as the catalytic subunit and a Cyclin regulatory subunit, either Cyclin T1 (CCNT1) or Cyclin T2 (CCNT2). P-TEFb activates transcriptional elongation by phosphorylating the carboxyl terminal domain (CTD) of the large subunit of RNAP II; P-TEFb also phosphorylates specific protein subunits of NELF and DSIF, and this negates their inhibition of elongation. For cellular genes, P-TEFb can be recruited to the RNAP II complex by different mechanisms to stimulate elongation. The bromodomain protein BRD4 is bound to a portion of P-TEFb and directs P-TEFb to actively transcribed genes that are marked by acetylated histones [7-9]. Transcription factors that bind directly to DNA elements in cellular promoters, such as NF-κB, cMyc, and MEF2, can also recruit P-TEFb to active genes [10-12].

P-TEFb is known to exist in three distinct complexes: 1) the “Core” complex composed of CDK9/CCNT1 or CCNT2/BRD4; 2) the “7SK snRNP” complex composed of CDK9/CCNT1 or CCNT2/7SK snRNA/ HEXIM1 or HEXIM2/LARP7/ MEPCE; 3) “Super Elongation Complex” (SEC) composed of CDK9/CCNT1 or CCNT2/ELL2/AFF4/ENL/AF9 [4]. Gel filtration analyses of purified Flag-tagged CDK9 have confirmed that these three complexes exist as distinct biochemical entities [13]. CDK9 kinase activity is repressed in the 7SK snRNP, and this complex is not thought to directly activate transcriptional elongation. Rather, the 7SK snRNP may be a catalytically inactive pool of P-TEFb from which active P-TEFb can be recruited to function in either the Core or SEC P-TEFb complex to activate elongation [14]. Consistent with this idea, disruption of the 7SK snRNP by siRNA depletions of HEXIM1 results in increased levels of Tat activation of the viral LTR [15].

The HIV Tat protein binds directly to CCNT1 and thereby targets only CCNT1-containing P-TEFb complexes [16]. Tat is capable of utilizing the Core P-TEFb complex by displacing Brd4 from CDK9/CCNT1 [17]. Tat can trigger the release of CDK9/CCNT1 from the 7SK snRNP and utilize this P-TEFb complex [18-20]. Tat also associates with the SEC and stabilizes this P-TEFb complex to activate viral gene expression [21,22].

Nearly all cellular processes are carried out by multi-protein complexes [23,24]. A recent study using a high-throughput integrative mass spectrometry-based analysis described the human endogenous complexome – the set of multi-protein complexes in HeLa cells [25]. In this landmark study, 1,796 primary antibodies were used in 3,390 immunoprecipitations of HeLa cell nuclear extracts. Conditions of immunoprecipitations were developed to preserve weak protein interactions and high levels of reciprocity in affinity purifications were used to identify distinct multi-protein complexes. We have mined this data set to identify multi-protein complexes that contain CDK9 and CCNT1. The three known P-TEFb complexes -- Core, 7SK snRNP and SEC -- as well as nine additional CDK9/CCNT1 complexes were identified in this analysis. In this study, we focused on five of these novel complexes and found that siRNA depletion of protein subunits of these novel complexes enhanced HIV-1 Tat function and viral gene expression. Although the mechanisms whereby siRNA disruption of these novel CDK9/CCNT1 complexes stimulate Tat function remain to be elucidated, our data suggest that their disruption may increase the level of P-TEFb available for Tat to utilize, similar to disruption of the 7SK snRNP. Our study indicates that the complexity of P-TEFb is considerably greater than previously appreciated.

## Results

### Identification of novel CDK9/CCNT1-associated proteins (CCAPs)

**Figure 1:**
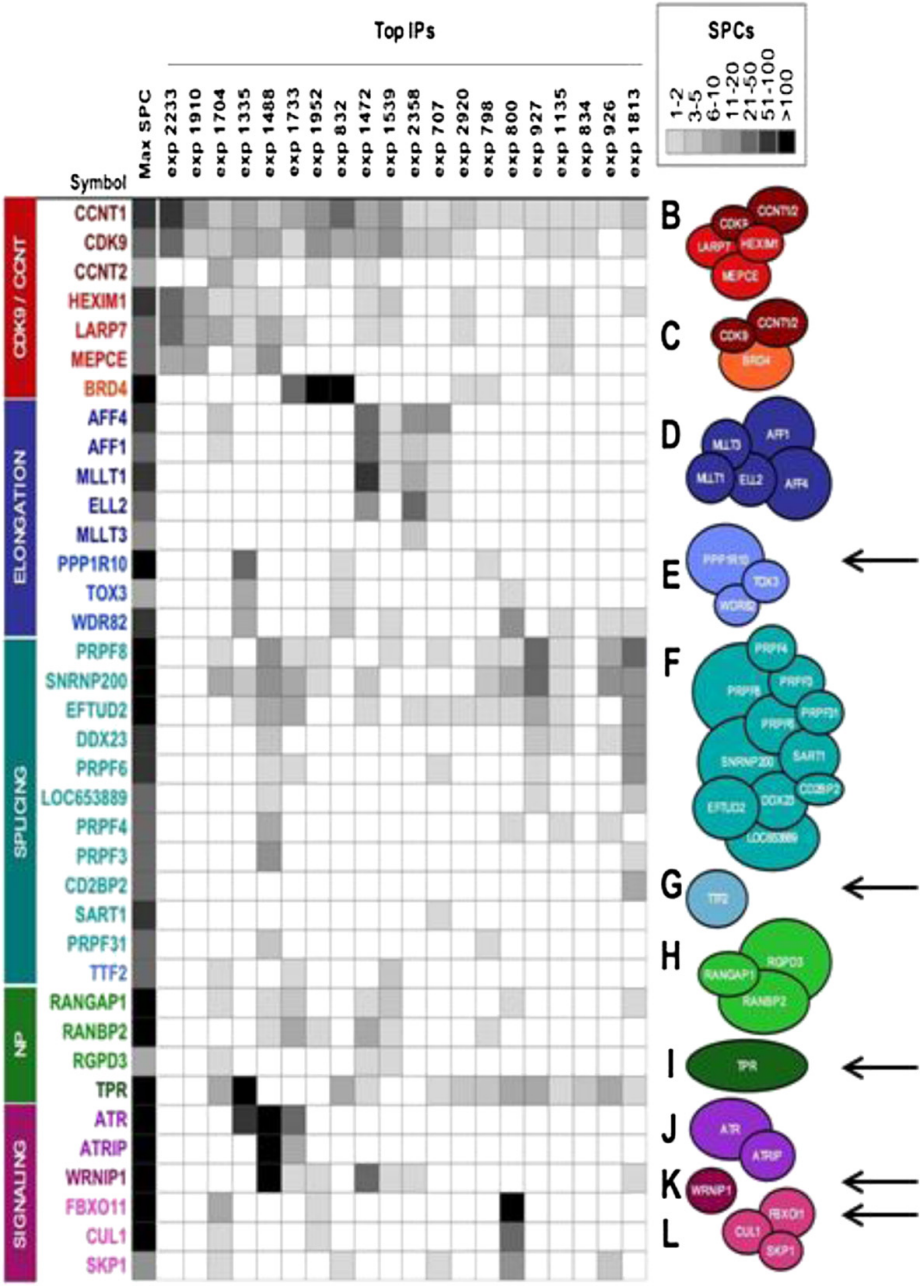
**CCAPs identified from human complexome.** The indicated CCAPs were derived from an integrative mass spectrometry analysis of a large scale immunoprecipitation study [25]. The range of spectral counts (SPCs; number of peptides identified by mass spectrometry) for proteins in CCAPs is indicated by a gray scale. Antisera used for each immunoprecipitation experiment are indicated at the top of the figure and can be found in Supplemental Information of reference 25. Arrows indicate the five CCAPs investigated in this study.

We mined the recently described human complexome dataset [25] to identify distinct protein complexes that contain both CDK9 and CCNT1. As shown in Figure 1, this analysis identified previously reported and novel CDK9/CCNT1-containing complexes. We refer to these complexes as CCAPs, for CDK9/CCNT1-associated protein complexes. Two CCAPs are mutually exclusive in our data set -- HEXIM1/LARP7/MEPCE (complex B) and BRD4 (complex C). The identification of these mutually exclusive CCAPs agrees precisely with traditional experimental approaches that identified these two distinct CDK9/CCNT1 complexes [26]. Additionally, the recently identified Super-elongation complex (SEC) complex (AFF4/AFF1/MLLT1/ELL2/MLLT3/; complex D [21,22,27]) was identified in our analysis, further validating this experimental approach to identify CCAPs of likely relevance to cellular and HIV-1 gene expression. 

A prominent CCAP not shown in Figure 1 due to size constraints contains CDK9/CCNT1 in a very large multiprotein complex that regulates transcription initiation and chromatin remodeling [CBP/p300; the 15 subunit INO80 complex; 20 subunit SWI/SNF complex; 30 subunit Mediator (Mediator has been implicated in HIV-1 replication in siRNA screens [28]). Two CCAPs involved in transcription elongation are prominent in Figure 1: AFF4/AFF1/MLLT1/ELL2/MLLT3 (Super-Elongation complex D) and WDR82/PPP1R10/TOX3 (complex E) that interacts with the LEO1/PAF complex (histone methyltransferase activity) and is implicated in transcriptional elongation [25]. TTF2 (complex G) is a CCAP involved in RNA Polymerase II termination. Two CCAPs associate with nuclear pores, RANGAP1 (complex H) and TPR (complex I). Other CCAPs included the checkpoint module ATR/ATRIP (complex J), ubiquitin binding protein WRNIP1 (complex K), and SCF^FBXO11^ ubiquitin E3 ligase (complex L). The proteins PRPF8 (complex F) and RANBP2 (complex H) have been implicated in HIV-1 replication in siRNA screens [28]. The identification of novel CCAPs involved in RNA elongation, RNA splicing, transcription termination, and nuclear pores suggest that the role of CDK9/CCNT1 in cellular and HIV-1 gene expression is considerably more intricate than previously appreciated.

### SiRNA depletion of CCAPs

To begin to investigate if CCAPs are involved in HIV-1 Tat function, we used siRNAs to deplete protein subunits of various CCAPs. Our preliminary functional assays for the effects of depletions of protein subunits of the five CCAPs indicated by arrows in Figure 1 suggested that these CCAPs negatively regulate Tat function. We therefore focused on these five CCAPs in this study. The properties of protein subunits of these five CCAPs are summarized in Table 1.

**Table 1:** Protein subunits of CCAPs depleted in this study

**Protein depleted**	**CCAP**	**Name**	**Other names**	**Function of depleted protein**	**Reference**
FBXO11	FBXO11	F-box protein 11	UBR6;VIT1; FBX11; PRMT9; UG063H01	Phosphorylation-dependent ubiquitination, regulates p53 and BCL6	[29,30]
	CUL1	Cullin 1	-		
	SKP1	S-phase kinase-associated protein 1	OCP2; p19A; EMC19; SKP1A; OCP-II; TCEB1L		
PPP1R10		protein phosphatase 1, regulatory subunit 10	FB19; CAT53; PNUTS; PP1R10	Targets protein phosphatase-1 (PP1) to the nucleus, in DNA damage response	[31]
WDR82	PNUTS	WD repeat domain 82	SWD2; MST107; WDR82A; MSTP107; PRO2730; TMEM113; PRO34047	Component of the mammalian SET1A /SET1B histone H3-Lys4-methyltransferase complexes	[32]
TOX3		TOX high mobility group box family member 3	CAGF9; TNRC9	Unwinding of DNA, chromatin structure alteration and neuronal transcription	[33]
TPR	TPR	Translocated promoter region (to activated MET oncogene)		Directly interacts with several components of nuclear pore complexes (NPCs). Nuclear export of mRNAs and some proteins.	[34]
TTF2	TTF2	Transcription termination factor, RNA polymerase II	HuF2	Critical role in altering protein-DNA interactions. Has dsDNA-dependent ATPase and RNAPII termination activity. Plays a role in pre-mRNA splicing.	[35,36]
WRNIP1	WRNIP1	Werner helicase interacting protein 1	WHIP; bA420G6.2; RP11-420G6.2	Interacts with the N-terminal portion of Werner protein containing the exonuclease domain and implicated in Werner’s syndrome. It may influence aging process.	[37-39]

The proteins that were depleted using siRNA in this study are listed. The core complex these proteins exist in cells as presented in Figure 1 is included along with brief description of their cellular function.

We used the TZM-bl cell line for our depletion experiments; this cell line is a HeLa cell derivative that expresses CD4, CXCR4, and CCR5 on its cell surface and therefore can be infected by HIV-1 [40]. TZM-bl cells also contain an integrated copy of the HIV-1 LTR with a Luciferase reporter protein. Tat transactivation assays can be readily performed in TZM-bl cells by transfection of a Tat expression plasmid and measurement of Luciferase expression from the integrated provirus. As the human complexome data set was generated in HeLa cells [25], the CCAPs shown in Figure 1 are likely similar if not identical between TZM-bl and HeLa cells.

**Figure 2:**
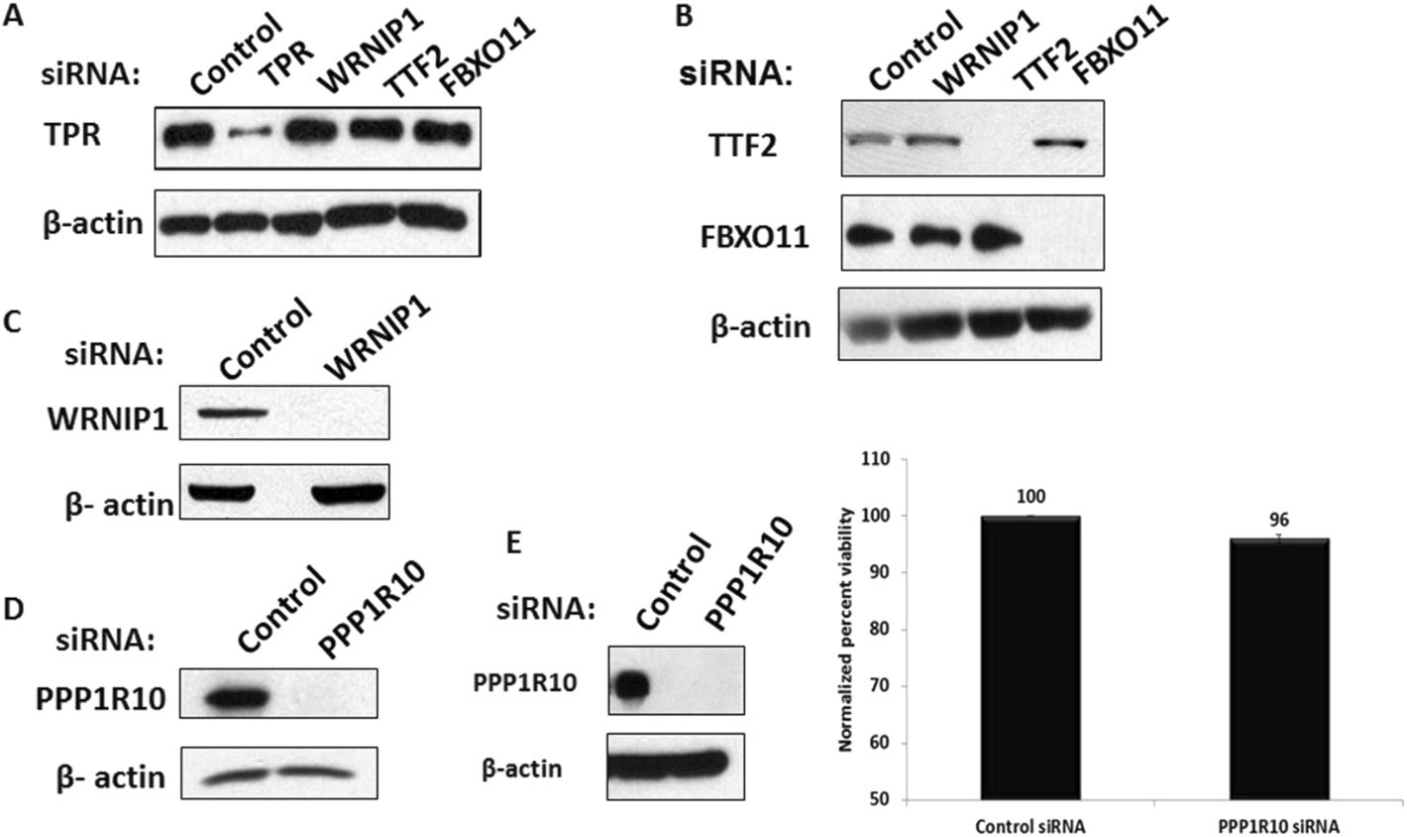
**SiRNA depletions of protein subunits of CCAPs.** TZM-bl cells were transfected with control siRNA or siRNAs against indicated proteins for 72 hours. Cell lysates were prepared, and protein expression was analysed by immunoblots. β-actin was used as a loading control. **(A)** Panel shows expression of TPR in cells transfected with control or siRNA against TPR, WRNIP1, TTF2 and FBXO11; **(B)** Top and middle panels show expression level of TTF2 and FBXO11, respectively, in cells transfected with control or siRNA against WRNIP1, TTF2 or FBXO11; **(C** and **D)** Panels show expression of WRNIP1 and PPP1R10 in cells transfected with siRNAs against WRNIP1 and PPP1R10, respectively. **(E)** Graph shows cell viability following transfection of control or PP1R10 siRNAs; the data are the average of three independent experiments with viability of control normalized to 100.

In the experiment shown in Figure 2A and 2B, TZM-bl cultures were transfected with siRNAs directed against either TTF2 (Figure 1, complex G), TPR (complex I), WRNIP1 (complex K), or FBXO11 (subunit of complex L). Cell lysates were prepared 72 hours after siRNA transfections, and the degrees of protein depletion were analysed by immunoblots. SiRNAs against TPR was effective in depleting TPR but had no effect on WRNIP1, TTF2, or FBXO11 (Figure 2A). SiRNAs against TTF2 effectively depleted TTF2 but had no effect on WRNIP1 or FBX011 (Figure 2B); likewise, siRNAs against FBXO11 were effective against FBXO11 but had no effect on WRNIP1 or TTF2 (Figure 2B). SiRNAs transfected in TZM-bl cells against WRNIP1 (complex K) and PPP1R10 (complex E) were also effective in depleting the target protein (Figure 2C, 2D). These data indicate that siRNA depletions are effective for the targeted subunits of these CCAPs.

Because PPR1R10 is associated with Protein Phosphatase 1 and regulates the activity of a cellular enzyme that regulates many cellular processes, we monitored the effect of siRNA depletion of PPP1R10 on cell viability using a Trypan Blue exclusion assay. TZM-bl cells were transfected with siRNAs against PPP1R10 or control siRNAs. Cells were collected at 72 hours post-transfection and divided into two groups -- one group was lysed, and depletion of PPP1R10 was verified by immunoblot (data not shown); and the other group was assayed for cell viability. As shown in Figure 2E, there was only a small reduction in the viability of the cells depleted of PPP1R10 compared to control siRNA-treated cells. This small reduction in cell viability by siRNA depletion of PP1R10 is unlikely to explain the enhancement of Tat function by the depletion (see below).

### SiRNA depletion of CCAPs enhances Tat activation of HIV-1 LTR

**Figure 3:**
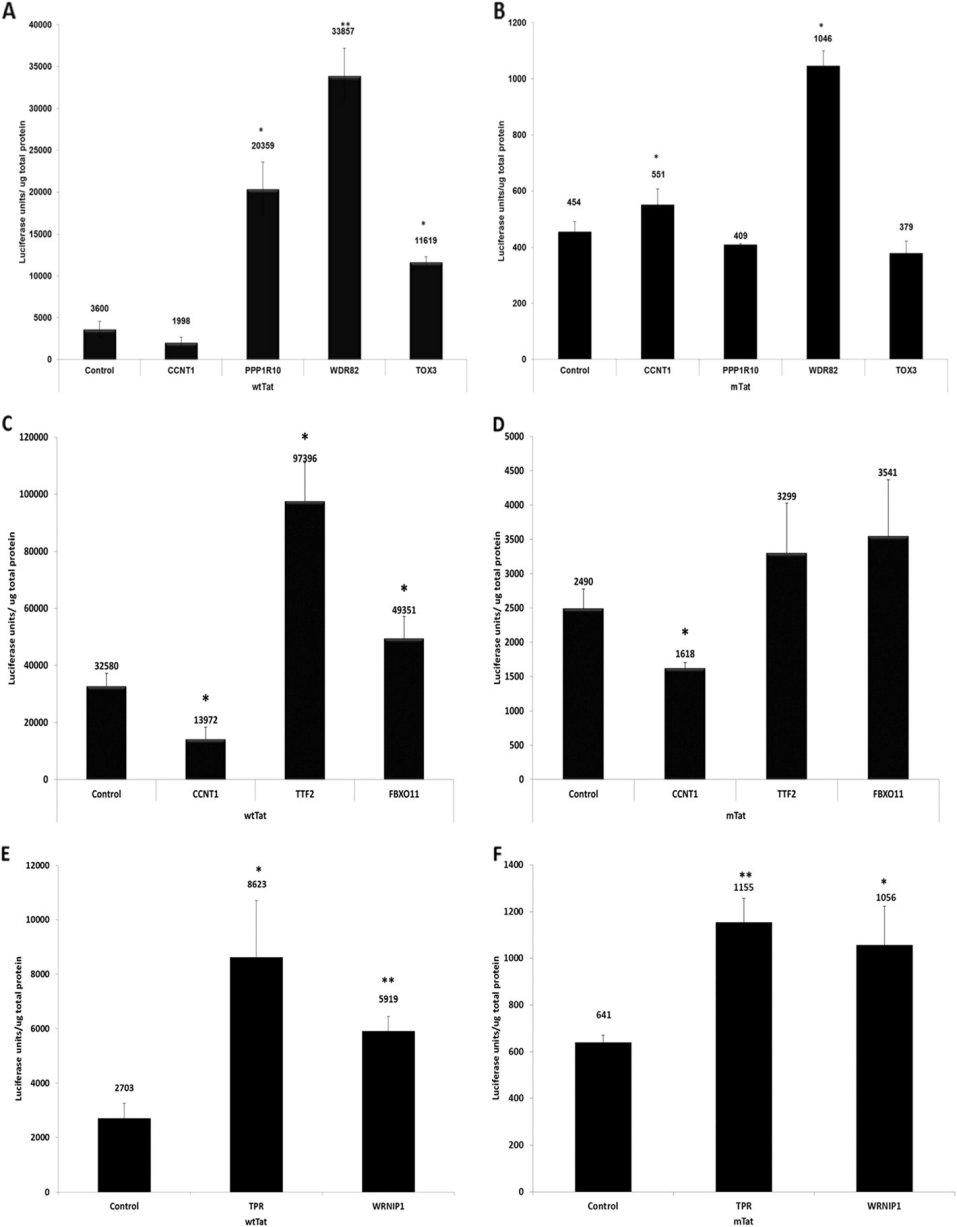
**Effects of siRNA depletions of CCAPs on Tat transactivation.** TZM-bl cells were transfected in triplicate with control siRNA or indicated siRNAs. 48 hours later, cells were transfected with expression plasmid for wild-type Tat (wtTat) **(A**, **C** and **E)** or transactivation-defective mutant Pro18IS Tat (mTat) **(B**, **D** and **F)**. Luciferase expression from the integrated HIV-1 LTR was measured 24 hours post-plasmid transfection and normalized to total cellular protein. A representative experiment is shown from three independent experiments. Statistical significance was estimated using Student’s t-test. (* p≤ 0.05 and ** p< 0.005). Note the difference in scales between panels **A**, **C**, **E** and **B**, **D**, **F**.

To examine the functional effects of depletions of protein subunits of CCAPs, TZM-bl cells were transfected with siRNAs, followed by transfection of expression plasmids for either wild type Tat (wtTat) or the transactivation-defective mutant termed Pro18IS Tat (mTat) [41]. Cell lysates were prepared 24 hours after Tat plasmid transfections and analysed for Luciferase expression from the integrated HIV-1 LTR in TZM-bl cells. Additionally, siRNAs against CCNT1 were transfected as a control in which Tat function should be reduced. When compared with transfection of control siRNAs, the depletion of CCNT1 inhibited activation of the integrated HIV-1 LTR Luciferase reporter by the wtTat protein as expected (Figure 3A, C). Depletion of subunits of each CCAP examined --- TPR, WRNIP1, TTF2, FBOX11, PPP1R10, WDR82, TOX3 – increased activation by the wtTat protein that was statistically significant in a Student’s T-test (Figure 3A, C, E). Depletion of the individual subunits of the PPP1R10 complex -- PPP1R10, WDR82 and TOX3 – also increased activation by wt Tat to a statistically significant level, from approximately 3- to 10-fold (Fisgure 3A). TTF2 and FBXO11 depletion activated the HIV-1 LTR Luciferase reporter by approximately 3- and 2-fold, respectively (Figure 3C), while depletion of TPR and WRNIP1 resulted in activation by approximately 3- and 2-fold, respectively, relative to control siRNAs (Figure 3E). We obtained similar results as those shown with wt Tat in Figure 3 with siRNA depletions of CCAPs in Jurkat T cells infected with an HIV-1 luciferase reporter virus, although the magnitude of Tat enhancement was less than that seen in TZM-bl cells (data not shown), likely due to inefficient siRNA transfections and depletions in Jurkat cells. In cells transfected with transactivation-defective mTat plasmid, depletion of TPR, WRNIP1, TTF2, FBXO11, and WDR82 also resulted in an induction HIV-1 LTR-driven Luciferase expression, although the level of Luciferase expression was considerably less than cells transfected with wtTat (Figure 3B, D, F). These data suggest that disruption of the five newly identified CCAPs by siRNA depletions enhances Tat activation of the HIV-1 LTR, perhaps by increasing the pool of CDK9/CCNT1 that is available for Tat function. 

Based on the results shown in Figure 3, it is conceivable that the five CCAPs examined could function additively to regulate Tat function. Therefore, we evaluated the effects of depletions of combinations of CCAPs on Tat transactivation in TZM-bl cells (Additional file 1: Figure S1). This analysis demonstrated that some combinations showed a strong additive effect on increasing Tat transactivation, especially the combination of TTF2 and FBXO11.

### Activation of NF-κB is not responsible for the increase of HIV-1 gene expression upon depletion of CCAPs

**Figure 4:**
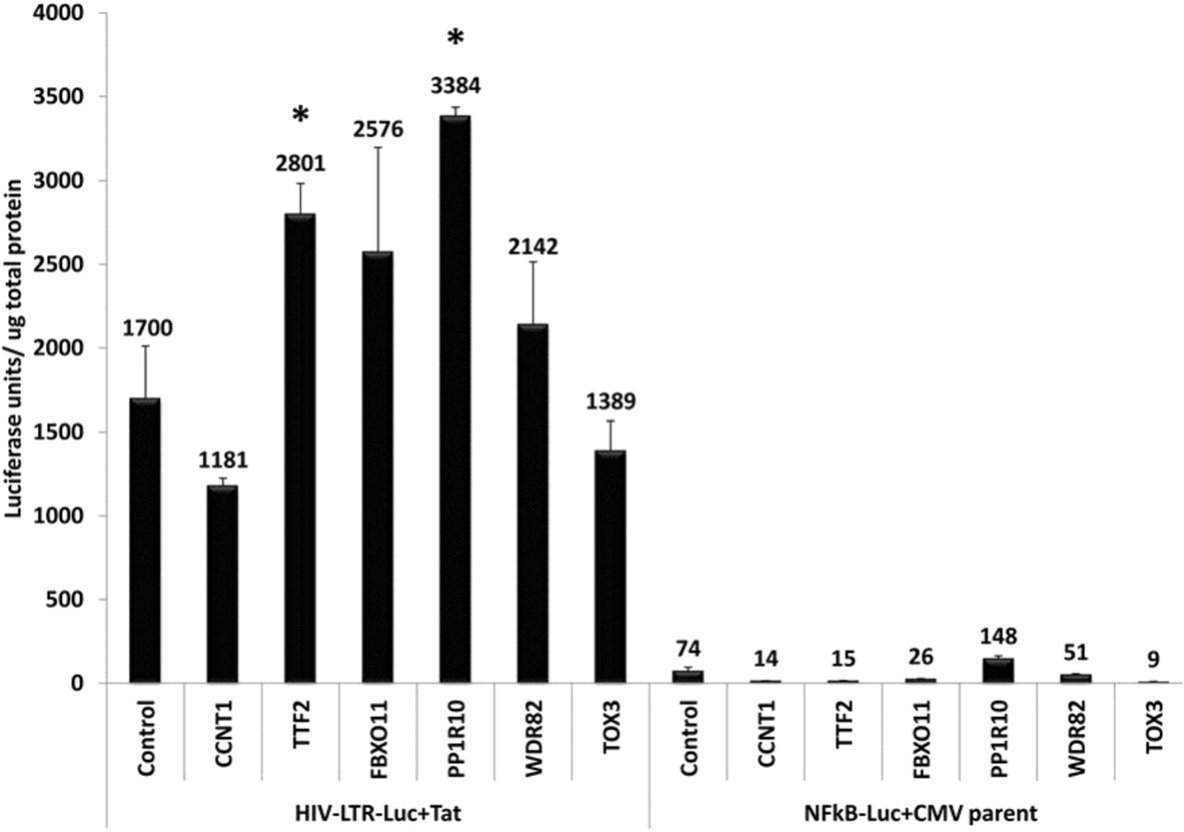
**Enhancement of Tat activation by CCAP depletions is not due to activation of NF-κB.** HeLa cells were transfected for 48 hours with control siRNA or the indicated siRNAs. Cells were then co-transfected with 500 ng HIV-1 LTR-Luciferase reporter plasmid plus 50 ng wtTat plasmid or 500 ng NF-κB-Luciferase reporter plasmid plus 50 ng pCMV parent vector. Cell extracts were prepared 24 hours post-plasmid transfection, and Luciferase expression was normalized to total cellular protein in cell extracts. Statistical significance was determined using a t-test. A representative experiment from three independent experiments is shown (* represents p value ≤0.05).

It is possible that the enhancement of viral gene expression by siRNA depletions of CCAPs may involve activation of NF-κB arising from a stress response to the depletions. To investigate this possibility, we carried out plasmid transfection assays with HIV-1 LTR and NF-κB Luciferase reporter plasmids in HeLa cells. Cultures were transfected with control or specific siRNAs, and 48 hours later cells were co-transfected a HIV-LTR Luciferase reporter plasmid plus a wtTat plasmid Tat or a NF-κB Luciferase reporter plasmid plus the CMV parental vector. Cell lysates were prepared 24 hours post-plasmid transfection, and Luciferase expression was measured (Figure 4). As expected, depletion of CCNT1 reduced expression of the HIV-1 LTR, while depletion of CCAPs (TTF2, FBXO11, PPP1R10, and WDR82,) enhanced viral LTR expression. Depletion of CCNT1 reduced expression from the NF-κB reporter, while depletion of all CCAPs other than PPP1R10 also reduced expression of the NF-κB reporter. These data indicate that depletion of CCAPs does not increase HIV LTR-directed gene expression through activation of NF-κB with the possible exception of PPP1R10.

### Enhancement of Tat activation by CCAP depletions is not the result of increased Tat protein expression or increased release of CDK9/CCNT1 from 7SK snRNP

**Figure 5:**
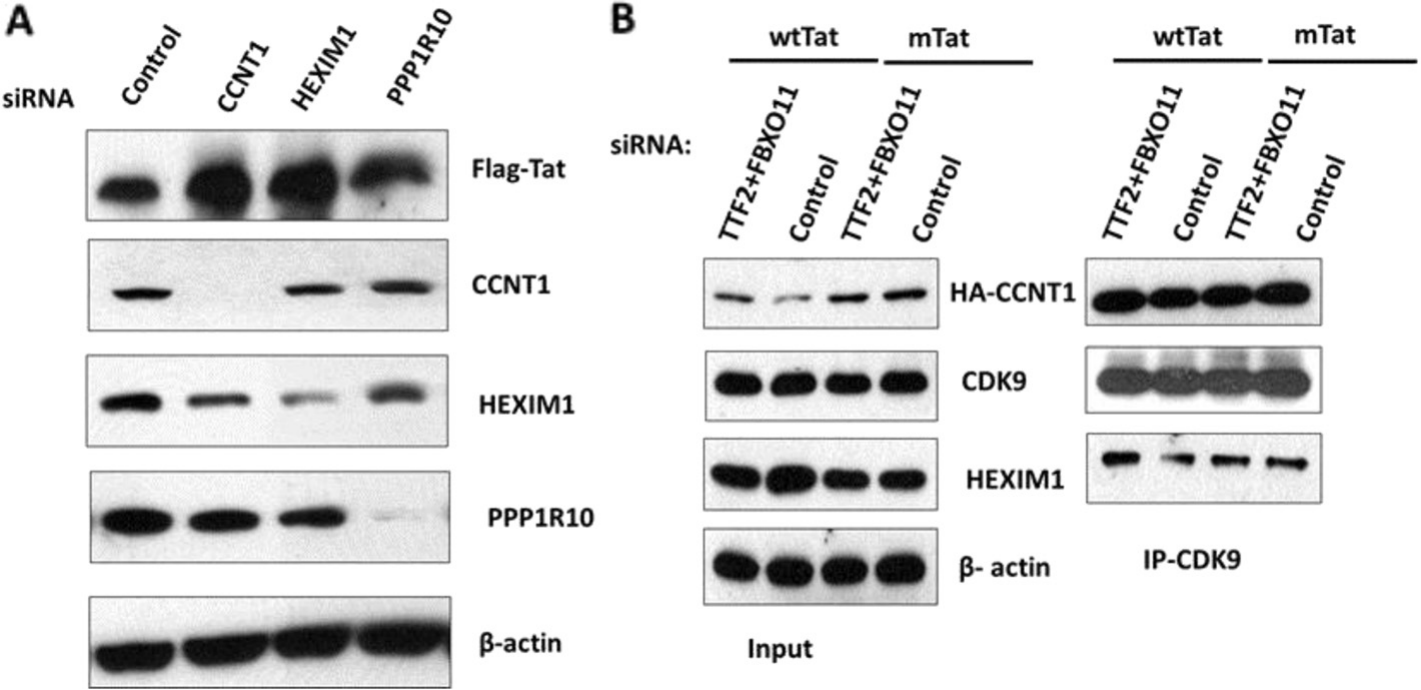
**Enhancement of Tat activation by CCAP depletions is not due to increased Tat expression or differential association of CDK9/CCNT1 with 7SK/HEXIM1.(A)** HeLa cells were transfected with indicated siRNAs for 48 hours followed by transfection of Flag-tagged wild type HIV-1 Tat expression plasmid. At 24 hours post-plasmid transfection, cell lysates were prepared and immunoblot analysis carried out for expression levels of Flag-Tat, CCNT1, HEXIM1 and PPP1R10; β-actin was probed as loading control. **(B)** HeLa cells were transfected with control or TTF2+ FBXO11 siRNAs and 48 hours later transfected with expression plasmids for HA-CCNT1 or Flag-wild type Tat (wtTat) or mutant Tat (mTat) for a further 48 hours. Cell lysates were prepared and immunoprecipitated with CDK9. Left part of the panel shows input levels, and right part shows the immunoprecipitated proteins.

It is possible that siRNA depletions of CCAPs results in increased Tat protein levels from the Tat expression plasmid, and this might contribute to increased Tat function in TZM-bl cells. To investigate this possibility, we transfected HeLa cells with control siRNA or siRNA against CCNT1, HEXIM1, or PPP1R10, followed by transfection of the Flag-tagged wild type HIV-1 Tat expression plasmid. PPPR1R10 was used in this control experiment as its depletion resulted in relatively high levels of enhancement of Tat activation (see Figure 3A and Figure 4). Cell lysates were prepared 24 hours post-plasmid transfection, and protein levels were analysed by immunoblot (Figure 5A). SiRNAs were effective in depletion of CCNT1 and PPP1R10, while depletion of HEXIM1 was somewhat less efficient. We did not observe a significant difference in the expression of Flag-Tat upon between the PPP1R10 depletion and control siRNA-treated cells (Figure 5A), although Tat activity was enhanced under these conditions of PPP1R10 depletion (Figures 3A, 4). This result suggests that depletion of CCAPs enhances Tat function through mechanisms other than increasing Tat levels from the plasmid vector.

We also investigated whether depletion of CCAPS might affect the amount of CDK9/CCNT1 in the 7SK snRNP. Depletion of TTF2 and FBXO11 was used for this control experiment because this combination of CCAP depletions showed the greatest level of Tat enhancement observed in this study (see Additional file 1: Figure S1). HeLa cells were first transfected with a combination of TTF2 and FBXO11 siRNAs or control siRNAs, and 48 hours later cells were transfected with expression plasmids for Flag-tagged wild type Tat or mutant Tat and HA-CCNT1; 48 hours later cell extracts were prepared for immunoprecipitations with an antiserum against CDK9. As shown in Figure 5B, no difference in the association of CDK9 with CCNT1 or HEXIM1 was observed in cells transfected with Flag-tagged wild type and mutant Tat and treated with either control or TTF2+ FBXO11 siRNAs. This result suggests that depletion of CCAPs does not affect the level of CDK9/CCNT1 in the 7SK snRNP.

### Combination of TTF2 and FBXO11 depletion sensitizes Jurkat CD4^+^ T cells to reactivation of latent HIV-1

**Figure 6:**
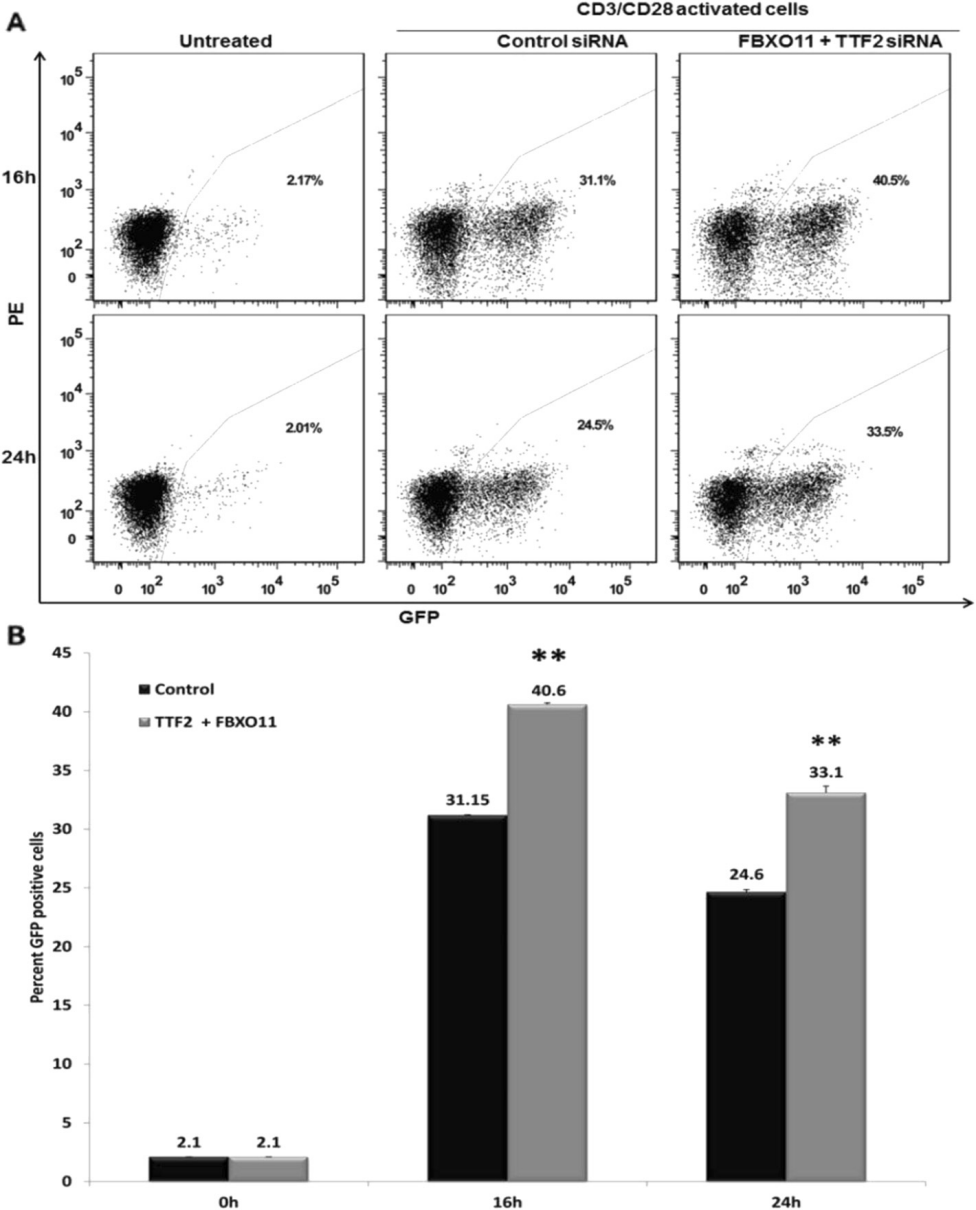
**Depletions of TTF2 and FBXO11 sensitize latent provirus in Jurkat 2D10 cell line to re-activation.** Duplicate Jurkat 2D10 cultures were transfected with control siRNA or siRNA against TTF2 and FBXO11 for 36 hours. Cells were then activated with suboptimal amounts (2μl) of CD3/CD28 beads and GFP expression from re-activated provirus was analysed by flow cytometry. A representative experiment from two independent experiments is shown. **(A)** The panel shows GFP expression in untreated cells (background GFP expression) and in activated cells treated with either control siRNA or TTF2 and FBXO11 siRNA at the indicated time points. The x-axis is GFP, and the y-axis is PE that is used as a placeholder fluorophore to enable visualization as a dot plot. A representative flow cytometric analysis of GFP expression from the duplicate samples is shown. **(B)** The graph represents average GFP expression in cells that were transfected with indicated siRNA. The untreated cells represent background GFP expression. Statistical significance was computed using the t-test (* represent p≤ 0.05 and ** represents p< 0.005).

The results presented above indicate that depletion of CCAPs increases Tat function and HIV-1 gene expression. We wished to determine if depletions of CCAPs would have an effect on reactivation of a latent HIV-1 provirus. We used the Jurkat 2D10 cell line for this experiment; 2D10 cells contain a latent provirus virus with a destabilized eGFP in place of Nef [42]. When treated with αCD3/CD28 beads, activation of the latent provirus can be examined by flow cytometry. We evaluated depletion of TTF2 + FBXO11 in this experiment, as this combination of depletion has the strongest enhancement of Tat activation in TZM-bl cells (Additional file 1: Figure S1). 2D10 cells were transfected with control siRNA or siRNAs against TTF2 and FBXO11. Thirty six hours post-siRNA transfection, cells were treated with sub-optimal amounts of αCD3/CD28 beads, and GFP expression was monitored by flow cytometry 16 and 24 hours later. Depletion of both TTF2 and FBXO11 resulted in an increase in the number of GFP+ cells relative to the control siRNA (Figure 6). At 16 hours post-activation, the TTF2/FBXO11 depletion demonstrated 34% GFP+ cells, and the control depletions demonstrated 27%. At 24 hours post-activation, 27% of cells depleted for both TTF2 and FBXO11 remained GFP+, while only 21% of cells transfected with control siRNAs were GFP+. These differences at 16 and 24 hour post-activation between the TTF2/FBXO11 and control siRNA depletions were statistically significant as determined by a t-test (p values < 0.005 and 0.05, respectively). This result is consistent with the data presented in Figures 3, 4 and Additional file 1: Figure S1 that indicate that depletion of the five CCAPs evaluated in this study enhances HIV-1 gene expression. Similar to depletion of HEXIM1 in the 7SK snRNP, it is possible that depletion of these CCAPs enhances viral gene expression by increasing the levels of CDK9/CCNT1 available for Tat function. 

## Discussion

The production of cellular mRNA and HIV-1 RNA from the integrated provirus is a highly regulated process in which the individual steps are linked. RNA splicing is coupled to transcriptional elongation [43], and nuclear export of processed RNA can be affected by splicing [44]. Most, if not all proteins responsible for production of RNA exist in multi-protein complexes, and the understanding of mechanisms involved in HIV-1 RNA production will require the identification of protein complexes involved in the individual steps of viral RNA production. In this study, we mined the recently described HeLa cell nuclear complexome [25] to investigate multi-protein complexes involved in HIV-1 Tat function and viral gene expression. Our analysis of the complexome data set identified 12 multi-protein complexes which contain CDK9/CCNT1, the enzymatic core of the general RNAP II elongation factor known as P-TEFb that mediates Tat transactivation. Our analysis identified the three previously known CDK9/CCNT1 complexes –Core, 7SK snRNP, and SEC P-TEFb complexes [4], thereby validating our analytical approach. We identified nine additional multi-protein complexes that contain CDK9/CCNT1; eight of these novel CDK9/CCNT1 complexes are shown in Figure 1, while the largest complex identified (not shown in Figure 1) contains CBP/p300, the 15 subunit INO80 complex, 20 subunit SWI/SNF complex, and 30 subunit Mediator complex. We have termed these multi-protein complexes CCAPs for CDK9/CCNT1-associated protein complexes.

To begin to investigate the role of novel CCAPs in Tat function, we carried out siRNA depletions of protein subunits of five CCAPs. We chose to focus on these five CCAPs in the present study as our initial results indicated that depletions of their protein subunits enhanced Tat function. We found that disruption of these CCAPs by siRNA depletions resulted in enhanced Tat function in TZM-bl cells. Because previous work has shown that depletion of HEXIM1 in the 7SK snRNP enhances Tat function by increasing the available pool of CDK9/CCNT1, we favour the hypothesis that depletion of these five CCAPs enhances Tat function by also increasing the available pool of CDK9/CCNT1. However, future studies will be required to determine the molecular mechanisms whereby disruption of these five CCAPs enhances Tat function. Our initial results with a CCAP containing ATR/ATRIP (Complex J, Figure 1) indicated that depletion of these proteins inhibits Tat function, consistent with a previous study [45]. Additionally, it is likely that depletion of subunits of the largest CCAP (CBP/p300, the 15 subunit INO80 complex, 20 subunit SWI/SNF complex, and 30 subunit Mediator complex) will inhibit Tat, as some of these protein subunits have been shown to play a positive role in Tat function and HIV-1 replication [28,46,47].

### CCAPs examined in this study

The protein subunits of the CCAPs examined here have been previously shown to be involved in a number of cellular processes of interest to cellular and HIV-1 RNA metabolism. The finding that these proteins associate with CDK9/CCNT1 has a number of intriguing functional and mechanistic implications. Short descriptions of the known functions of the protein subunits of these CCAPs are presented below.

### PPP1R10/TOX3/WDR82

Complex E (Figure 1) recruits the phosphatase PP1 into the nucleus and is involved in the DNA damage response [31,48]. Additionally, WDR82 is found in the SET1A histone H3-Lys4-methyltransferase complex [32] and this chromatin modification in associated with actively expressed genes.

### TTF2

This CCAP subunit is involved in the termination of RNAP II transcription [35]. TTF2 has also been implication in mitotic repression of transcriptional elongation [36]. A recent publication reported that TTF2 is associated with decapping factors and the Xrn2 exonuclease at transcriptional start sites and is involved in premature termination of RNAP II transcription [49].

### TPR

This CCAP subunit is a large coiled-coiled protein and is localized within the nuclear basket of the nuclear pore complex; TPR plays a role in nuclear export of mRNA [34,50]. Fusion of the 5′ end of the TPR gene with several different kinase genes is associated with cancer.

### WRNIP1

This CCAP subunit interacts with the Werner protein and is implicated in Werner’s syndrome [37]. The Werner protein is involved in RNAP II transcription and its dysfunction in Werner’s syndrome results in an accelerated aging phenotype [38].

### FBXO11/CUL1/SKP1

This CCAP is involved in phosphorylation-dependent ubiquitylation of target proteins and has been shown to regulated BCL6 and p53 [29,30].

### Complexity of P-TEFb and CCAPs

The identification of 12 CCAPs in this study and the heterogeneity in the catalytic core of P-TEFb reveal an enormous potential complexity in the biological functions of CDK9 and CCNT1. The catalytic core is composed of a heterodimer of CDK9 and a Cyclin subunit, either CCNT1 or CCNT2 [3]. Additionally, two spliced variants of CCNT2 are expressed, CCNT2a and CCNT2b [51], as are two isoforms of CDK9, a major 42 kDa isoform and a minor 55 kDa isoform that arises from an upstream transcriptional start site [52]. It is possible that these two CDK9 isoforms may differentially associate with the CCAPs shown in Figure 1, and it is possible that one or both of the CCNT2 isoforms may replace CCNT1 in these multi-protein complexes. Complexes that differ by their CDK9 or Cyclin subunit may have distinct functional properties, as well as distinct expression patterns in different tissues. Transcriptional profiling of cells depleted for either CCNT1 or CCNT2 has identified mRNAs whose expression patterns appear dependent on either CCNT1 or CCNT2 [53,54]. The expression pattern of the 42 kDa and 55 kDa CDK9 proteins varies across different murine tissues, with liver having higher expression of the 55 kDa protein than the 42 kDa protein [55]. The 55 kDa, but not 42 kDa, CDK9 protein has also been shown to accumulate in the nucleolus of HeLa cells [56].

### Regulation of in CCAPs in CD4^+^ T lymphocytes and monocytes/macrophages

The expression patterns of the protein subunits of CCAPs in CD4^+^ T cells and monocytes/macrophages have important implications for HIV-1 replication, as these are the two major cell types infected by HIV-1 *in vivo*. Resting CD4^+^ T cells do not support HIV-1 replication, in part because P-TEFb function is limiting [57,58]. In resting CD4^+^ T cells, CCNT1 levels are low, and this involves repression of translation of CCNT1 mRNA by miRNAs [59]. Upon T cell activation, this repression is released and there is a strong induction of CCNT1 protein levels [60-62]. In resting CD4^+^ T cells, the catalytic function of CDK9 is further repressed by dephosphorylation of T186 in the T-loop of the kinase; upon T cell activation there is a rapid induction of T-loop phosphorylation and CDK9 kinase activity [63,64]. Also in resting CD4^+^ T cells, the level of the 7SK snRNP complex is low, and upon T cell activation there is a large increase the level of the 7SK snRNP [65,66]. In preliminary experiments, we found that the protein subunits of the five CCAPs investigated in this study are expressed at low level in resting CD4^+^ T-cells and are induced upon cellular activation, suggesting that the levels of these CCAPs are up-regulated following T cell activation. Thus, it appears that resting CD4^+^ T cells require only very low levels of CCAPs to support basal metabolism in quiescent T cells.

Similarly, CDK9 and CCNT1 are highly regulated in monocytes and macrophages. In monocytes, miR-198 represses expression of CCNT1 [67] and although the 42 kDa CDK9 protein is generally expressed at a high level, the CDK9 T-loop is not phosphorylated [68]. Upon macrophage differentiation, CCNT1 levels are strongly up-regulated and the CDK9 T-loop is phosphorylated [68-70]. The 55 kDa CDK9 protein is also up-regulated during macrophage differentiation [56]. Similar to resting and activated CD4^+^ T cells, the 7SK snRNP complex is expressed at only low levels in quiescent monocytes and it is induced following macrophage differentiation [71]. Upon extended time in culture, CCNT1 expression is shut-off by proteasome-mediated proteolysis [69]. However, CCNT1 but not CCNT2 expression can be up-regulated in late differentiated macrophage by either HIV-1 infection or activation with pathogen-associated molecular patterns (PAMPs). With the exception of Core P-TEFb and the 7SK snRNP, the expression patterns of CCAPs shown in Figure 1 have not been examined in monocytes and macrophages.

### Future studies

Four of the novel CCAPs identified by our analysis remain to be investigated for potential roles in Tat function (Figure 1, complexes F, H, J, and the CBP/p300, the 15 subunit INO80 complex, 20 subunit SWI/SNF complex, and 30 subunit Mediator complex). Our preliminary results with the ATR/ATRIP CCAP suggest that it may act to positively regulate Tat function and viral gene expression, in agreement with a previous study [45]. ATR is a protein kinase involved in the DNA damage response, and it is activated by the HIV-1 Vpr protein [72]. The finding that ATR/ATRIP is found in a complex with CDK9/CCNT1 raises the intriguing possibility of a mechanistic link between Tat and Vpr functions. Complex H contains RNAGAP1/RANBP2/RFPD3 and is involved in nuclear pore function, suggesting that Tat activation of RNAP II elongation may be coupled to RNA export and perhaps HIV-1 Rev function via this complex. In summary, our identification of 12 multi-protein complexes which contain CDK9/CCNT1 reveals considerable biological complexity in this cellular kinase and will serve as the basis for future research on molecular mechanisms involved in HIV-1 gene expression and replication.

## Conclusions

Our results identified a very large potential complexity in the biological functions of CDK9 and CCNT1. Our results suggest that at least five newly identified multi-protein complexes contain both CDK9 and CCNT1 and may reduce the amount of these proteins available for Tat function.

## Methods

### Cell culture and activation

TZM-bl, HeLa, and 293T cells were cultured in DMEM supplemented with 10% fetal bovine serum (FBS) and 1% penicillin-streptomycin. Jurkat CD4^+^ T cells and Jurkat 2D10 cells were cultured in RPMI supplemented with 10% fetal bovine serum (FBS) and 1% penicillin-streptomycin. 2D10 cells were activated using Dynabeads CD3/CD28 T cell expander beads (Invitrogen) according to the manufacturer’s protocol.

### SiRNA and plasmid transfections

Control and siRNAs against individual subunits of CCAPs were purchased from Santa Cruz Biotechnology, CA as pools of three target specific siRNAs. The pool of three siRNAs was specific for distinct target sites in the transcript of the protein to be depleted. This approach has an advantage over using individual siRNAs since depletion of target protein could be achieved with lower amount of siRNA reducing the risk of off-target effects. SiRNAs (10–30 pmol) was delivered into TZM-bl, HeLa, Jurkat or 2D10 cells by either a reverse or traditional transfection method. For reverse transfections, TZM-bl or HeLa cells were seeded in 10 cm plates at 24 hours before experiment. On the day of transfection, the cells were collected by trypsinization and suspended in DMEM with 10% FBS and no antibiotics (50,000 cells/ well/sample) in 24 well tissue culture plates. Transfection was carried out using Lipofectamine RNAiMAX (Invitrogen) according to the manufacturer’s protocol. SiRNAs and Lipofectamine RNAiMAX were diluted in Opti-MEM1 and mixed together. The siRNA-transfection reagent mixture was incubated at room temperature (RT) for 20 minutes and added to the cells. For traditional transfections, the protocol used was as described above except that TZM-bl or HeLa cells were seeded in 24 well tissue culture plates (50,000 cells/well/sample) at 18 hours before experiments. Jurkat cells and 2D10 cells were seeded the same day of experiment and transfected with siRNAs as described above.

In experiments with TZM-bl cells in 24 well tissue culture plates, cells were transfected for 48 hours with 50 ng of pFLAG- wild type HIV-1 Tat (wtTat) or pFLAG-pro18IS-HIV-1 Tat (mTat) using Lipofectamine 2000 (Invitrogen) according to the manufacturer’s instructions. In other plasmid transfection experiments carried out in HeLa cells, 500 ng HIV-1 LTR reporter plasmid and 50 ng Tat expression plasmid were transfected as described for TZM-bl cells. In these experiments, cells were also transfected with 500 ng pNF-kB-Luc and 50 ng pcDNA empty vector (pCMV parent) ensuring equal amounts of DNA were transfected.

### Immunoblot and immunoprecipitation analysis

Cells were lysed with EBCD buffer (50mM Tris–HCl, pH 8.0, 120mM NaCl, 0.5% NP-40, 5mM dithiothreitol) containing protease inhibitor cocktail (Sigma). Immunoblotting was performed as described previously [4]. TTF2, FBXO11, WRNIP1, TPR, PPP1R10, Hsp70 and β-actin were probed using antibodies against TTF2 (Abcam, 1:1000), FBXO11 (Bethyl Labs, 1:2000), WRNIP1 (Santa Cruz, 1:1000), TPR (Santa Cruz, 1: 1000), PPP1R10 (Abcam, 1: 1000), and β-actin (Sigma) (1:5000), respectively. Immunoblots were quantified using Image J software [73]. Immunoprecipitation was carried out as described previously [65,74].

### Luciferase reporter assays

Cells were washed with sterile PBS and cell lysates prepared with Cell Culture Lysis Buffer (Promega). Cell lysates were analyzed for Luciferase activity using Luciferase Assay Kit (Promega) according to manufacturers’ protocol. Luciferase assay products were measured using a luminometer (Turner). Total protein in the lysates was estimated using a Bradford assay (Bio-Rad) and used to normalize the Luciferase readings.

### Flow cytometry analysis

2D10 cells were examined for GFP expression on a BD Fortessa flow cytometer. 10,000 events were collected and the data analysed using BD FACS Diva software. For visualization of the data as a dot plot, PE was used as a placeholder fluorophore.

## Competing interests

The authors declare that they have no competing interests.

## Authors’ contributions

RR, HL, HD carried out experiments and analysed the data; APR, AM and JQ analysed data; RR and APR wrote the manuscript. All authors read and approved the final manuscript.

## Supplementary Material

Additional file 1 **Figure S1.** Depletion of combinations of CCAPs has an additive effect on HIV-1 Tat function in TZM-bl cells. TZM-bl cells were transfected with control siRNA or combination of the indicated siRNAs for 48 hours. Cells were then transfected with HIV-1 Tat (wtTat) and 24 hours later cell lysates were prepared and examined for Luciferase expression. Luciferase expression values were normalized to amount of total cellular protein. A representative experiment from three independent experiments is shown. Statistical significance was estimated using t-test. (* p≤ 0.05 and ** p< 0.005). (PDF 159 kb) Click here for file

## References

[B1] Karn J Stoltzfus CM Transcriptional and Posttranscriptional Regulation of HIV-1 Gene Expression Cold Spring Harb Perspect Med 2012 2 a006916 22355797 10.1101/cshperspect.a006916 PMC3281586

[B2] He N Zhou Q New Insights into the Control of HIV-1 Transcription: When Tat Meets the 7SK snRNP and Super Elongation Complex (SEC) J Neuroimmune Pharmacol 2011 6 260 8 10.1007/s11481-011-9267-6 21360054 PMC3087102

[B3] Peterlin BM Price DH Controlling the elongation phase of transcription with P-TEFb Mol Cell 2006 23 297 305 10.1016/j.molcel.2006.06.014 16885020

[B4] Ott M Geyer M Zhou Q The Control of HIV Transcription: Keeping RNA Polymerase II on Track Cell Host Microbe 2011 10 426 435 10.1016/j.chom.2011.11.002 22100159 PMC3478145

[B5] Guenther MG Levine SS Boyer LA Jaenisch R Young RA A chromatin landmark and transcription initiation at most promoters in human cells Cell 2007 130 77 88 10.1016/j.cell.2007.05.042 17632057 PMC3200295

[B6] Glover-Cutter K Kim S Espinosa J Bentley DL RNA polymerase II pauses and associates with pre-mRNA processing factors at both ends of genes Nat Struct Mol Biol 2008 15 71 78 10.1038/nsmb1352 18157150 PMC2836588

[B7] Jang MK Mochizuki K Zhou M Jeong HS Brady JN Ozato K The bromodomain protein Brd4 is a positive regulatory component of P-TEFb and stimulates RNA polymerase II-dependent transcription Mol Cell 2005 19 523 534 10.1016/j.molcel.2005.06.027 16109376

[B8] Yang Z Yik JH Chen R He N Jang MK Ozato K Zhou Q Recruitment of P-TEFb for stimulation of transcriptional elongation by the bromodomain protein Brd4 Mol Cell 2005 19 535 545 10.1016/j.molcel.2005.06.029 16109377

[B9] Hargreaves DC Horng T Medzhitov R Control of inducible gene expression by signal-dependent transcriptional elongation Cell 2009 138 129 145 10.1016/j.cell.2009.05.047 19596240 PMC2828818

[B10] Barboric M Nissen RM Kanazawa S Jabrane-Ferrat N Peterlin BM NF-kappaB binds P-TEFb to stimulate transcriptional elongation by RNA polymerase II Mol Cell 2001 8 327 337 10.1016/S1097-2765(01)00314-8 11545735

[B11] Eberhardy SR Farnham PJ Myc recruits P-TEFb to mediate the final step in the transcriptional activation of the cad promoter J Biol Chem 2002 277 40156 40162 10.1074/jbc.M207441200 12177005

[B12] Nojima M Huang Y Tyagi M Kao HY Fujinaga K The positive transcription elongation factor b is an essential cofactor for the activation of transcription by myocyte enhancer factor 2 J Mol Biol 2008 382 275 287 10.1016/j.jmb.2008.07.017 18662700 PMC4118929

[B13] Smith E Lin C Shilatifard A The super elongation complex (SEC) and MLL in development and disease Genes Dev 2011 25 661 672 10.1101/gad.2015411 21460034 PMC3070929

[B14] Dow EC Liu H Rice AP T-loop phosphorylated Cdk9 localizes to nuclear speckle domains which may serve as sites of active P-TEFb function and exchange between the Brd4 and 7SK/HEXIM1 regulatory complexes J Cell Physiol 2010 224 84 93 20201073 10.1002/jcp.22096 PMC2888102

[B15] Yik JH Chen R Nishimura R Jennings JL Link AJ Zhou Q Inhibition of P-TEFb (CDK9/Cyclin T) kinase and RNA polymerase II transcription by the coordinated actions of HEXIM1 and 7SK snRNA Mol Cell 2003 12 971 982 10.1016/S1097-2765(03)00388-5 14580347

[B16] Tahirov TH Babayeva ND Varzavand K Cooper JJ Sedore SC Price DH Crystal structure of HIV-1 Tat complexed with human P-TEFb Nature 2010 465 747 751 10.1038/nature09131 20535204 PMC2885016

[B17] Bisgrove DA Mahmoudi T Henklein P Verdin E Conserved P-TEFb-interacting domain of BRD4 inhibits HIV transcription Proc Natl Acad Sci USA 2007 104 13690 13695 10.1073/pnas.0705053104 17690245 PMC1959443

[B18] Schulte A Czudnochowski N Barboric M Schonichen A Blazek D Peterlin BM Geyer M Identification of a cyclin T-binding domain in Hexim1 and biochemical analysis of its binding competition with HIV-1 Tat J Biol Chem 2005 280 24968 24977 10.1074/jbc.M501431200 15855166

[B19] Barboric M Yik JH Czudnochowski N Yang Z Chen R Contreras X Geyer M Matija PB Zhou Q Tat competes with HEXIM1 to increase the active pool of P-TEFb for HIV-1 transcription Nucleic Acids Res 2007 35 2003 12 10.1093/nar/gkm063 17341462 PMC1874611

[B20] Sedore SC Byers SA Biglione S Price JP Maury WJ Price DH Manipulation of P-TEFb control machinery by HIV: recruitment of P-TEFb from the large form by Tat and binding of HEXIM1 to TAR Nucleic Acids Res 2007 35 4347 4358 10.1093/nar/gkm443 17576689 PMC1935001

[B21] He N Liu M Hsu J Xue Y Chou S Burlingame A Krogan NJ Alber T Zhou Q HIV-1 Tat and host AFF4 recruit two transcription elongation factors into a bifunctional complex for coordinated activation of HIV-1 transcription Mol Cell 2010 38 428 438 10.1016/j.molcel.2010.04.013 20471948 PMC3085314

[B22] Sobhian B Laguette N Yatim A Nakamura M Levy Y Kiernan R Benkirane M HIV-1 Tat assembles a multifunctional transcription elongation complex and stably associates with the 7SK snRNP Mol Cell 2010 38 439 451 10.1016/j.molcel.2010.04.012 20471949 PMC3595998

[B23] Alberts B The cell as a collection of protein machines: preparing the next generation of molecular biologists Cell 1998 92 291 294 10.1016/S0092-8674(00)80922-8 9476889

[B24] O’Malley BW Qin J Lanz RB Cracking the coregulator codes Curr Opin Cell Biol 2008 20 310 315 10.1016/j.ceb.2008.04.005 18499426 PMC3647352

[B25] Malovannaya A Lanz RB Jung SY Bulynko Y Le NT Chan DW Ding C Shi Y Yucer N Krenciute G Analysis of the human endogenous coregulator complexome Cell 2011 145 787 799 10.1016/j.cell.2011.05.006 21620140 PMC3131083

[B26] Michels AA Bensaude O RNA-driven cyclin-dependent kinase regulation: when CDK9/cyclin T subunits of P-TEFb meet their ribonucleoprotein partners Biotechnol J 2008 3 1022 1032 10.1002/biot.200800104 18655042

[B27] Benedikt A Baltruschat S Scholz B Bursen A Arrey TN Meyer B Varagnolo L Muller AM Karas M Dingermann T The leukemogenic AF4-MLL fusion protein causes P-TEFb kinase activation and altered epigenetic signatures Leukemia 2011 25 135 144 10.1038/leu.2010.249 21030982

[B28] Bushman FD Malani N Fernandes J D’Orso I Cagney G Diamond TL Zhou H Hazuda DJ Espeseth AS Konig R Host cell factors in HIV replication: meta-analysis of genome-wide studies PLoS Pathog 2009 5 e1000437 10.1371/journal.ppat.1000437 19478882 PMC2682202

[B29] Duan S Cermak L Pagan JK Rossi M Martinengo C di Celle PF Chapuy B Shipp M Chiarle R Pagano M FBXO11 targets BCL6 for degradation and is inactivated in diffuse large B-cell lymphomas Nature 2012 481 90 93 22113614 10.1038/nature10688 PMC3344385

[B30] Abida WM Nikolaev A Zhao W Zhang W Gu W FBXO11 promotes the Neddylation of p53 and inhibits its transcriptional activity J Biol Chem 2007 282 1797 1804 17098746 10.1074/jbc.M609001200 PMC3690493

[B31] Landsverk HB Mora-Bermudez F Landsverk OJ Hasvold G Naderi S Bakke O Ellenberg J Collas P Syljuasen RG Kuntziger T The protein phosphatase 1 regulator PNUTS is a new component of the DNA damage response EMBO Rep 2010 11 868 875 10.1038/embor.2010.134 20890310 PMC2966950

[B32] Wu M Wang PF Lee JS Martin-Brown S Florens L Washburn M Shilatifard A Molecular regulation of H3K4 trimethylation by Wdr82, a component of human Set1/COMPASS Mol Cell Biol 2008 28 7337 7344 10.1128/MCB.00976-08 18838538 PMC2593441

[B33] Dittmer S Kovacs Z Yuan SH Siszler G Kogl M Summer H Geerts A Golz S Shioda T Methner A TOX3 is a neuronal survival factor that induces transcription depending on the presence of CITED1 or phosphorylated CREB in the transcriptionally active complex J Cell Sci 2011 124 252 260 10.1242/jcs.068759 21172805

[B34] Frosst P Guan T Subauste C Hahn K Gerace L Tpr is localized within the nuclear basket of the pore complex and has a role in nuclear protein export J Cell Biol 2002 156 617 630 10.1083/jcb.200106046 11839768 PMC2174070

[B35] Liu M Xie Z Price DH A human RNA polymerase II transcription termination factor is a SWI2/SNF2 family member J Biol Chem 1998 273 25541 25544 10.1074/jbc.273.40.25541 9748214

[B36] Jiang Y Liu M Spencer CA Price DH Involvement of transcription termination factor 2 in mitotic repression of transcription elongation Mol Cell 2004 14 375 385 10.1016/S1097-2765(04)00234-5 15125840

[B37] Kanamori M Seki M Yoshimura A Tsurimoto T Tada S Enomoto T Werner interacting protein 1 promotes binding of Werner protein to template-primer DNA Biol Pharm Bull 2011 34 1314 1318 10.1248/bpb.34.1314 21804224

[B38] Balajee AS Machwe A May A Gray MD Oshima J Martin GM Nehlin JO Brosh R Orren DK Bohr VA The Werner syndrome protein is involved in RNA polymerase II transcription Mol Biol Cell 1999 10 2655 2668 10436020 10.1091/mbc.10.8.2655 PMC25497

[B39] Comai L Li B The Werner syndrome protein at the crossroads of DNA repair and apoptosis Mech Ageing Dev 2004 125 521 528 10.1016/j.mad.2004.06.004 15336909

[B40] Platt EJ Wehrly K Kuhmann SE Chesebro B Kabat D Effects of CCR5 and CD4 cell surface concentrations on infections by macrophagetropic isolates of human immunodeficiency virus type 1 J Virol 1998 72 2855 2864 9525605 10.1128/jvi.72.4.2855-2864.1998 PMC109730

[B41] Rice AP Carlotti F Mutational analysis of the conserved cysteine-rich region of the human immunodeficiency virus type 1 Tat protein J Virol 1990 64 1864 1868 2181156 10.1128/jvi.64.4.1864-1868.1990 PMC249332

[B42] Pearson R Kim YK Hokello J Lassen K Friedman J Tyagi M Karn J Epigenetic silencing of human immunodeficiency virus (HIV) transcription by formation of restrictive chromatin structures at the viral long terminal repeat drives the progressive entry of HIV into latency J Virol 2008 82 12291 12303 10.1128/JVI.01383-08 18829756 PMC2593349

[B43] Kornblihtt AR De la MM Fededa JP Munoz MJ Nogues G Multiple links between transcription and splicing RNA 2004 10 1489 1498 10.1261/rna.7100104 15383674 PMC1370635

[B44] Han J Xiong J Wang D Fu XD Pre-mRNA splicing: where and when in the nucleus Trends Cell Biol 2011 21 336 343 10.1016/j.tcb.2011.03.003 21514162 PMC6553873

[B45] Dehart JL Andersen JL Zimmerman ES Ardon O An DS Blackett J Kim B Planelles V The ataxia telangiectasia-mutated and Rad3-related protein is dispensable for retroviral integration J Virol 2005 79 1389 1396 10.1128/JVI.79.3.1389-1396.2005 15650165 PMC544104

[B46] Mahmoudi T Parra M Vries RG Kauder SE Verrijzer CP Ott M Verdin E The SWI/SNF Chromatin-remodeling Complex Is a Cofactor for Tat Transactivation of the HIV Promoter J Biol Chem 2006 281 19960 19968 10.1074/jbc.M603336200 16687403

[B47] Agbottah E Deng L Dannenberg LO Pumfery A Kashanchi F Effect of SWI/SNF chromatin remodeling complex on HIV-1 Tat activated transcription Retrovirology 2006 3 48 10.1186/1742-4690-3-48 16893449 PMC1570494

[B48] Lee JH You J Dobrota E Skalnik DG Identification and characterization of a novel human PP1 phosphatase complex J Biol Chem 2010 285 24466 24476 10.1074/jbc.M110.109801 20516061 PMC2915683

[B49] Brannan K Kim H Erickson B Glover-Cutter K Kim S Fong N Kiemele L Hansen K Davis R Lykke-Andersen J mRNA decapping factors and the exonuclease Xrn2 function in widespread premature termination of RNA polymerase II transcription Mol Cell 2012 46 311 324 10.1016/j.molcel.2012.03.006 22483619 PMC3806456

[B50] Coyle JH Bor YC Rekosh D Hammarskjold ML The Tpr protein regulates export of mRNAs with retained introns that traffic through the Nxf1 pathway RNA 2011 17 1344 1356 10.1261/rna.2616111 21613532 PMC3138570

[B51] Peng J Zhu Y Milton JT Price DH Identification of multiple cyclin subunits of human P-TEFb Genes Dev 1998 12 755 762 10.1101/gad.12.5.755 9499409 PMC316581

[B52] Shore SM Byers SA Maury W Price DH Identification of a novel isoform of Cdk9 Gene 2003 307 175 182 12706900 10.1016/s0378-1119(03)00466-9

[B53] Kohoutek J Li Q Blazek D Luo Z Jiang H Peterlin BM Cyclin T2 is essential for mouse embryogenesis Mol Cell Biol 2009 29 3280 3285 10.1128/MCB.00172-09 19364821 PMC2698739

[B54] Ramakrishnan R Yu W Rice AP Limited redundancy in genes regulated by Cyclin T2 and Cyclin T1 BMC Res Notes 2011 4 260 10.1186/1756-0500-4-260 21791050 PMC3160394

[B55] Shore SM Byers SA Dent P Price DH Characterization of Cdk9(55) and differential regulation of two Cdk9 isoforms Gene 2005 350 51 58 10.1016/j.gene.2005.01.015 15780980

[B56] Liu H Herrmann CH Differential localization and expression of the Cdk9 42k and 55k isoforms J Cell Physiol 2005 203 251 260 10.1002/jcp.20224 15452830

[B57] Rice AP Herrmann CH Regulation of TAK/P-TEFb in CD4+ T lymphocytes and macrophages Curr HIV Res 2003 1 395 404 10.2174/1570162033485159 15049426

[B58] Tyagi M Pearson RJ Karn J Establishment of HIV latency in primary CD4+ cells is due to epigenetic transcriptional silencing and P-TEFb restriction J Virol 2010 84 6425 6437 10.1128/JVI.01519-09 20410271 PMC2903277

[B59] Chiang K Sung TL Rice AP Regulation of Cyclin T1 and HIV-1 Replication by MicroRNAs in Resting CD4+ T Lymphocytes J Virol 2012 86 3244 3252 10.1128/JVI.05065-11 22205749 PMC3302325

[B60] Herrmann CH Carroll RG Wei P Jones KA Rice AP Tat-associated kinase, TAK, activity is regulated by distinct mechanisms in peripheral blood lymphocytes and promonocytic cell lines J Virol 1998 72 9881 9888 9811724 10.1128/jvi.72.12.9881-9888.1998 PMC110500

[B61] Garriga J Peng J Parreno M Price DH Henderson EE Grana X Upregulation of cyclin T1/CDK9 complexes during T cell activation Oncogene 1998 17 3093 3102 10.1038/sj.onc.1202548 9872325

[B62] Marshall RM Salerno D Garriga J Grana X Cyclin T1 expression is regulated by multiple signaling pathways and mechanisms during activation of human peripheral blood lymphocytes J Immunol 2005 175 6402 6411 16272292 10.4049/jimmunol.175.10.6402

[B63] Ghose R Liou LY Herrmann CH Rice AP Induction of TAK (cyclin T1/P-TEFb) in purified resting CD4(+) T lymphocytes by combination of cytokines J Virol 2001 75 11336 11343 10.1128/JVI.75.23.11336-11343.2001 11689614 PMC114719

[B64] Ramakrishnan R Dow EC Rice AP Characterization of Cdk9 T-loop phosphorylation in resting and activated CD4(+) T lymphocytes J Leukoc Biol 2009 86 1345 1350 10.1189/jlb.0509309 19741158 PMC2780919

[B65] Haaland RE Herrmann CH Rice AP Increased association of 7SK snRNA with Tat cofactor P-TEFb following activation of peripheral blood lymphocytes AIDS 2003 17 2429 2436 10.1097/00002030-200311210-00004 14600513

[B66] Haaland RE Herrmann CH Rice AP siRNA depletion of 7SK snRNA induces apoptosis but does not affect expression of the HIV-1 LTR or P-TEFb-dependent cellular genes J Cell Physiol 2005 205 463 470 10.1002/jcp.20528 16152622

[B67] Sung TL Rice AP miR-198 inhibits HIV-1 gene expression and replication in monocytes and its mechanism of action appears to involve repression of cyclin T1 PLoS Pathog 2009 5 e1000263 10.1371/journal.ppat.1000263 19148268 PMC2607557

[B68] Dong C Kwas C Wu L Transcriptional restriction of human immunodeficiency virus type 1 gene expression in undifferentiated primary monocytes J Virol 2009 83 3518 3527 10.1128/JVI.02665-08 19211771 PMC2663290

[B69] Liou LY Herrmann CH Rice AP Transient induction of cyclin T1 during human macrophage differentiation regulates human immunodeficiency virus type 1 Tat transactivation function J Virol 2002 76 10579 10587 10.1128/JVI.76.21.10579-10587.2002 12368300 PMC136632

[B70] Liou LY Herrmann CH Rice AP Human immunodeficiency virus type 1 infection induces cyclin T1 expression in macrophages J Virol 2004 78 8114 8119 10.1128/JVI.78.15.8114-8119.2004 15254183 PMC446126

[B71] Liou LY Haaland RE Herrmann CH Rice AP Cyclin T1 but not cyclin T2a is induced by a post-transcriptional mechanism in PAMP-activated monocyte-derived macrophages J Leukoc Biol 2006 79 388 396 16330531 10.1189/jlb.0805429

[B72] Roshal M Kim B Zhu Y Nghiem P Planelles V Activation of the ATR-mediated DNA damage response by the HIV-1 viral protein R J Biol Chem 2003 278 25879 25886 10.1074/jbc.M303948200 12738771

[B73] Abramoff MD Magalhaes PJ Ram SJ Image Processing with ImageJ Biophotonics International 2004 11 36 42

[B74] Budhiraja S Ramakrishnan R Rice AP Phosphatase PPM1A negatively regulates P-TEFb function in resting CD4T+ T cells and inhibits HIV-1 gene expression Retrovirology 2012 9 52 10.1186/1742-4690-9-52 22727189 PMC3406988

