# Supplementary figures and images for: Identification of novel CDK9 and Cyclin T1-associated protein complexes (CCAPs) whose siRNA depletion enhances HIV-1 Tat function
Source: Retrovirology. 2012 Oct 30;9:90. doi: 10.1186/1742-4690-9-90 (PMC3494656; doi:10.1186/1742-4690-9-90)

Supplemental Figure 1

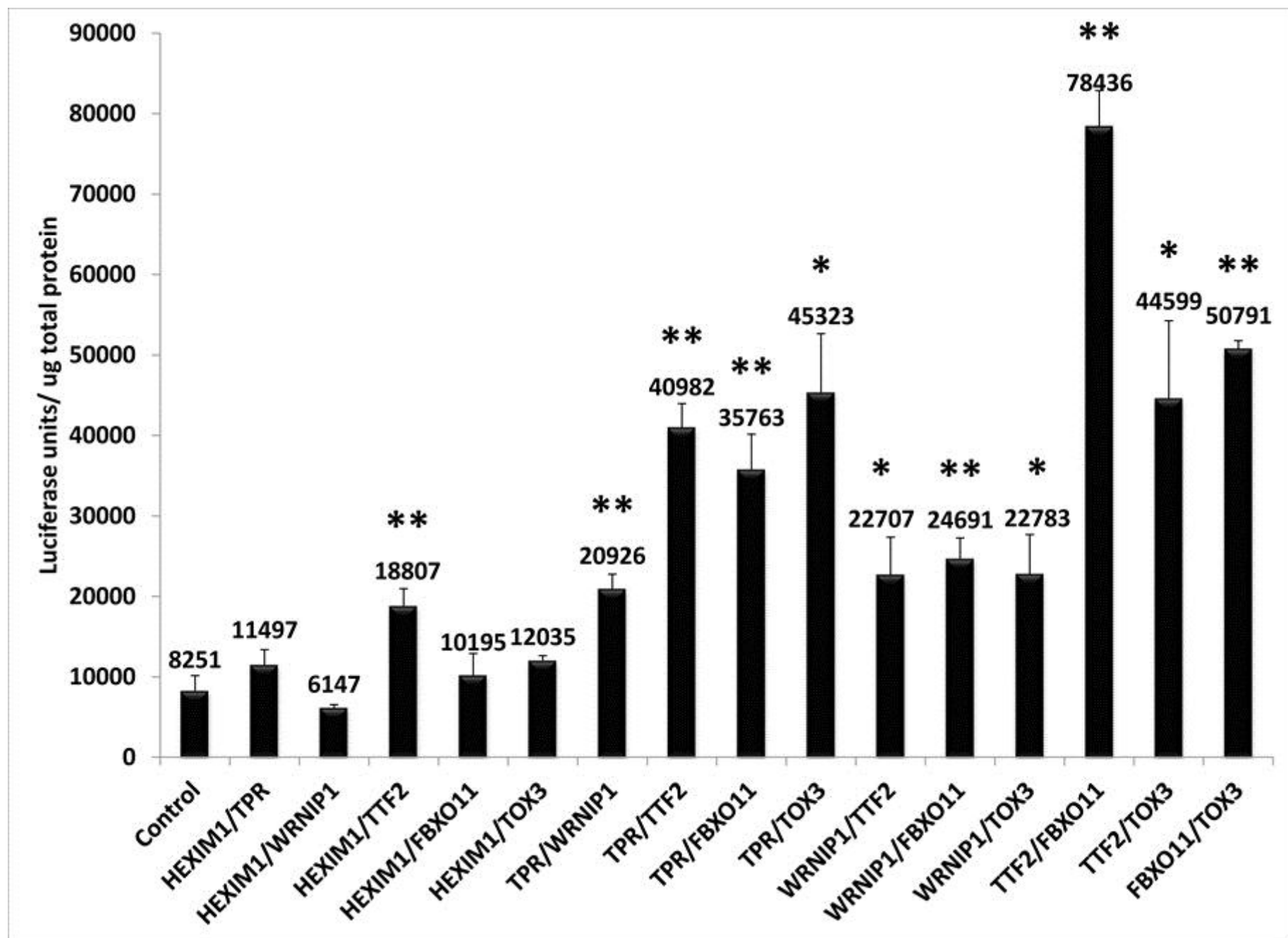

Supplement: Additional file 1 — Figure S1. Depletion of combinations of CCAPs has an additive effect on HIV-1 Tat function in TZM-bl cells. TZM-bl cells were transfected with control siRNA or combination of the indicated siRNAs for 48 hours. Cells were then transfected with HIV-1 Tat (wtTat) and 24 hours later cell lysates were prepared and examined for Luciferase expression. Luciferase expression values were normalized to amount of total cellular protein. A representative experiment from three independent experiments is shown. Statistical significance was estimated using t-test. (* p≤ 0.05 and ** p< 0.005). (PDF 159 kb) [file 1742-4690-9-90-S1.pdf]
